# Possible and Probable Dementia and Depressive/Anxiety Symptoms: Mediation Effects of Social Engagement and Loneliness

**DOI:** 10.1177/01640275251380531

**Published:** 2025-09-16

**Authors:** Namkee G. Choi, Yuanjin Zhou, C. Nathan Marti

**Affiliations:** 1 School of Social Work, 12330 The University of Texas at Austin, Austin, TX, USA

**Keywords:** dementia, social withdrawal, loneliness, depression, anxiety

## Abstract

Using a nationally representative sample of older adults (age 65+, *N* = 7,547) and a path model, we examined the direct effects of dementia on social engagement, loneliness, and depressive/anxiety symptoms, and the mediation effects of loneliness and social engagement on the associations between dementia and depressive/anxiety symptoms. Of the study population, 7.7% and 5.5% had possible and probable dementia, respectively; 14.3% and 21.9% of those with possible and probable dementia had moderate to severe depressive/anxiety symptoms. The ratios of the indirect effects of social engagement and loneliness on a possible dementia to the total effect of possible dementia on depressive/anxiety symptoms were 0.26 and 0.28, respectively. The ratios in probable dementia were 0.11 and 0.21. The lack of social engagement and loneliness contribute substantially to depressive/anxiety symptoms in dementia. Strategies to increase social engagement and reduce social isolation are necessary to improve the psychological well-being of people living with dementia.

## Introduction

The prevalence of depressive/anxiety symptoms among older adults with dementia, across all stages of the disease, is markedly higher than among their cognitively intact peers (Leung et al., 2021). Depression/anxiety exerts substantial adverse effects on individuals’ cognitive health and overall quality of life (Burks et al., 2021). Persistent or worsening depressive/anxiety symptoms are associated with accelerated dementia progression, affective and emotional dysregulation, and hastened functional decline (Botto et al., 2022; Defrancesco et al., 2017; Huang et al., 2022; Ismail et al., 2018; Lenze et al., 2005; Yin et al., 2024; Zhu et al., 2023). The comorbidity of dementia and depression/anxiety also complicates disease management, increases caregiver burden, and contributes to greater healthcare utilization (Chen et al., 2025; MacNeil-Vroomen et al., 2020; Xiang & An, 2015).

In addition to shared neurobiological mechanisms, depressive/anxiety symptoms among individuals with dementia likely reflect psychosocial responses to the awareness of cognitive decline (Botto et al., 2022; Brzezińska et al., 2020). In particular, the lack of social engagement and the experience of loneliness represent critical psychosocial stressors. During the early stages of dementia, individuals often feel shame, embarrassment, or frustration in social situations as cognitive, emotional, and functional impairments begin to interfere with their ability to maintain social roles and meaningful relationships (Aldridge et al., 2019; Honda et al., 2013; Kłosińska & Leszko, 2024; Singleton et al., 2017; Trindade et al., 2023). Dementia-related behavioral changes, such as apathy, disinhibition, and agitation, can further complicate interpersonal interactions and disrupt social integration (Hackett et al., 2019; Lozupone et al., 2024). Moreover, individuals with dementia may encounter social exclusion, discreditation, or discrimination in their social interactions (Biggs et al., 2019; Birt et al., 2020). Together, these challenges frequently lead to social withdrawal and reduced participation in community life, intensifying feelings of loneliness and depressive/anxiety symptoms and contributing to dementia progression (Penninkilampi et al., 2018; Wang et al., 2023).

Loneliness, defined as a subjective feeling of social disconnection or an unpleasant emotional experience resulting from a perceived deficiency in the quality of one’s social relationships (Perlman & Peplau, 1984), has increasingly been recognized as a critical biopsychosocial risk factor in later life. A substantial body of research has demonstrated its detrimental effects on the onset and progression of dementia (Htun et al., 2025; Lee et al., 2022, 2025; Luchetti et al., 2024; Oken et al., 2024; Rafnsson et al., 2020; Shen et al., 2022; Sundström et al., 2020), depression/anxiety (Lee et al., 2021), cardiovascular disease (Albasheer et al., 2024; Okeke et al., 2025), and increased risk of premature mortality (Holt-Lunstad et al., 2015; Wilson et al., 2019). Among individuals with dementia, loneliness may further precipitate or exacerbate depressive/anxiety symptoms by intensifying psychological distress, existential concerns, and feelings of worthlessness or hopelessness (Kotwal et al., 2024; Victor et al., 2020). Moreover, loneliness shares neurobiological and epigenetic mechanisms with both dementia and depression/anxiety, suggesting overlapping biological pathways that may heighten vulnerability to psychological decline (Bowirrat et al., 2023; Lam et al., 2021).

In contrast to the extensive body of research identifying a lack of social engagement and loneliness as risk factors for cognitive decline and dementia, their impact on the well-being of older adults already living with dementia remains relatively understudied. As discussed above, the limited existing studies, primarily based on convenience samples, suggest that reduced social engagement and heightened loneliness among individuals with dementia may contribute to increased depressive/anxiety symptoms. More population-based research is needed to examine the mediating role of social disengagement and loneliness in the relationship between dementia and depressive/anxiety symptoms. Such investigations could help disentangle the psychological pathways through which dementia affects mental health outcomes and identify key points of intervention.

Social health, including social participation and social networks, likely acts as a driver for the use of cognitive reserve, which can then slow cognitive impairment or maintain cognitive functioning (Vernooij-Dassen et al., 2022). Furthermore, depression/anxiety in dementia, whether they are a cause of cognitive impairment or a reaction to it, are important modifiable factors in dementia prevention, intervention, and care (Livingston et al., 2024). A better understanding of social engagement and loneliness as the mechanisms of the associations between dementia and depression/anxiety is essential for the development of targeted, person-centered interventions aimed at promoting social connection and emotional well-being among individuals with dementia.

**Figure 1.**
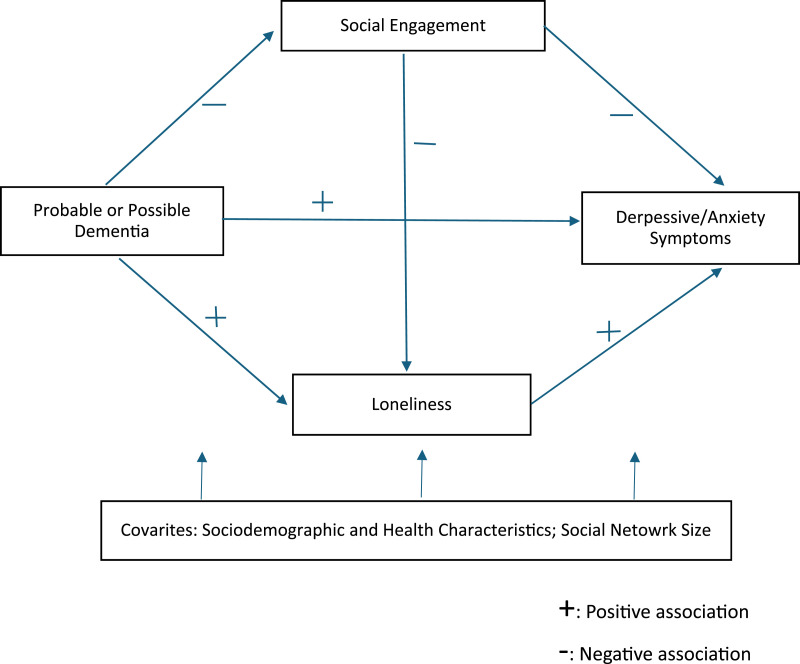
Conceptual and Analytic Framework for the Direct and Indirect Effects of Dementia, Social Engagement, and Loneliness on Depressive/Anxiety Symptoms

In this study, using a nationally representative sample of community-dwelling Medicare beneficiaries aged 65 and older, we examined the direct associations between dementia status (i.e., possible [less severe] or probable [more severe] dementia vs. no dementia) and social engagement (i.e., participation in social or community activities), loneliness, and depressive/anxiety symptoms. We then assessed the extent to which the relationship between dementia and depressive/anxiety symptoms was mediated by social engagement and loneliness. The study hypotheses were: (H1) depressive/anxiety symptoms would be positively associated with both possible and probable dementia and with loneliness, but negatively associated with social engagement; (H2a) dementia would be negatively associated with social engagement; (H2b) dementia would be positively associated with loneliness; (H3) social engagement would be negatively associated with loneliness; and (H4) the association between dementia and depressive/anxiety symptoms would be partially mediated by the influence of dementia on social engagement and loneliness. Given the higher psychosocial burden of probable than possible dementia, we further hypothesized that these mediation effects would be stronger for probable dementia than for possible dementia. Sociodemographic characteristics, chronic illness, activity-limiting pain, and social network size were included as covariates. Figure 1 shows the study’s conceptual and analytic framework. Findings are expected to provide new insights into the interrelationships among social engagement, loneliness, and depressive/anxiety symptoms among older adults living with dementia.

## Methods

### Data and Sample

Data came from the 2023 National Health and Aging Trends Study, which collects data annually from a nationally representative panel of Medicare beneficiaries ages 65 and older on their physical, functional, cognitive, and sensory capacities; social, physical, and technological environments; and participation in valued activities. In this study, after excluding 390 (of whom 16 had possible and 340 had probable dementia) sample persons who were proxy-interviewed, we focused on 7,547 sample persons who lived in their own homes or residential care communities (but not in nursing homes). The primary reason for including self-respondents only was that NHATS did not collect data on the sample persons’ loneliness (and other well-being indicators) from their proxy interviewees. This study, based on the analysis of de-identified public-use data, was exempt from the authors’ institutional review board review.

### Measures

*Dementia status* (no dementia, possible dementia, and probable dementia) was determined using the most recently updated NHATS dementia classification algorithm (Kasper et al., 2024) based on two types of information for self-respondents: (1) doctor diagnosis of dementia or AD (yes or no); and (2) scores from cognitive tests evaluating the sample person in the following three domains: memory (immediate and delayed 10-word recall), orientation (date, month, year, and day of the week; naming the President and Vice President), and executive function (clock drawing test). A possible dementia classification was assigned when the person scored ≤ 1.5 SD below the mean in one domain of the cognitive test. A probable dementia classification was assigned when the person was diagnosed with dementia or scored ≤1.5 standard deviations (SD) below the mean in at least two domains of the cognitive tests. Thus, possible dementia is a classification of less severe cognitive impairment than probable dementia. The score cut points for ≤1.5 SDs below the mean on NHATS cognitive domains were ≤3 for memory (score range 0 to 20), ≤3 for orientation (score range 0 to 8), and ≤1 for executive function (score range 0 to 5).

#### Depression/Anxiety Symptoms in the Past Month

In NHATS, depression/anxiety symptoms were assessed with the Patient Health Questionnaire-4 (PHQ-4) (Kroenke et al., 2009). The PHQ-4 includes the first two items (PHQ-2; had little interest or pleasure in doing things, and felt down, depressed, or hopeless) from the 9-item PHQ-9 for depression (Kroenke et al., 2003) and the first two items (GAD-2; felt nervous, anxious, or on edge, and have been unable to stop or control worrying) from the 7-item Generalized Anxiety Disorder Scale (Spitzer et al., 2006). Responses to each PHQ-4 item were based on a 4-point scale (0 = not at all; 1 = several days; 2 = more than half the days; 3 = nearly every day), with the total score ranging from 0 to 12. Depression and anxiety frequently coincide, with highly correlated symptoms, and act as mutual risk factors, regardless of age groups, resulting in comorbid depression and anxiety (Bian et al., 2024; Jacobson & Newman, 2017; King-Kallimanis et al., 2009). The unweighted Cronbach’s alpha for the PHQ-4 for the study sample was 0.77. The PHQ-4 scores were also used to categorize the symptom severity: no symptom (0-2), mild symptoms (3-5), and moderate/severe symptoms (6-12) for descriptive purposes (Kroenke et al., 2009).

#### Social Engagement

This was assessed by the number of the following six social or community activities that the sample persons participated in during the past month: a) visits with family/friends; (b) attendance in religious services; (c) participation in clubs, classes, or other organized activities aside from religious services (referred to as attendance in clubs/meetings hereafter); (d) working for pay; (e) volunteering; and (f) going out to dinner, a movie, gambling, or to hear music or see a play (i.e., going out for enjoyment). The number ranged from 0 to 6.

#### Loneliness

This was assessed with a question, “During the last month, how often did you feel lonely?” The response categories were every day (=5), most days (5–6 days a week) (=4), some days (2–4 days a week) (=3), rarely (once a week or less) (=2), and never (=1). We treated it as a continuous variable, with the score ranging from 1 to 5, and the higher score representing greater loneliness. For descriptive purposes, we also grouped them into three categories (never/rarely, some days, and five or more days).

*Covariates* included (1) sociodemographic factors: age (65–74 [reference], 75-84, 85+); gender (female vs. male); race/ethnicity (non-Hispanic White [reference], non-Hispanic Black, Hispanic, others); residential type (care community vs. own home); education (bachelor’s degree or higher vs. no degree); and income, defined as total annual income from all sources reported for the respondent and their spouse or partner (if any), including earnings, pensions, Social Security, retirement accounts, and other public or private benefits (<$43,000 vs. other); (2) health status: the number of chronic medical conditions (0-8: arthritis, cancer, hypertension, heart disease, stroke, diabetes, lung disease, osteoporosis), and activity-limiting chronic pain (yes or no); and (3) social network size: the number of people (up to five) with whom the sample person discussed important life matters, including positive or negative events and concerns. Social network size is considered a social environmental factor (Vernooij-Dassen et al., 2022) that may influence social engagement and help decrease loneliness among older adults with dementia.

### Analysis

All analyses were conducted with Stata/MP 19.5’s svy function (College Station, TX) to account for NHATS’s stratified, multistage sampling design. First, we used χ^2^ and one-way ANOVA to compare all study variables by dementia status: No dementia, possible dementia, and probable dementia. We then used χ^2^ and *t* tests to compare the two dementia groups.

Second, we fitted a path model to test hypotheses regarding the direct effects of possible and probable dementia, loneliness, and social engagement on depressive/anxiety symptoms, as well as the mediating roles of loneliness and social engagement in the association between dementia status and depressive/anxiety symptoms. Path coefficients (B) and linearized standard errors (SE) with 95% confidence intervals (CIs) were estimated using the survey-adjusted SEM framework. To test whether the associations of possible and probable dementia with social engagement and loneliness differed significantly in strength, we conducted adjusted Wald tests comparing the corresponding path coefficients. To assess mediation, we estimated the indirect effects of possible and probable dementia on depressive/anxiety symptoms through each mediator. Indirect effects and their 95% CIs were computed using bootstrapping with 10,000 replications. We also bootstrapped the differences in indirect effects across dementia status (i.e., probable vs. possible dementia) for each mediator to formally test whether the magnitude of mediation differed significantly. In addition, we calculated the proportion of the total effect that was mediated (indirect effect divided by the sum of direct and indirect effects), and the ratio of indirect to direct effects, for each pathway. Note that, given the cross-sectional data, while we use terms such as direct and indirect effects from the mediation literature, we do not infer causal relationships, but the findings reflect associations.

## Results

### Prevalence of Depressive/Anxiety Symptoms, Social Engagement, and Loneliness

Table 1 shows that among community-dwelling Medicare beneficiaries aged 65 and older who were self-respondents (i.e., not proxy interviewed), 7.7% and 5.5% were classified as having possible and probable dementia, respectively, in 2023. Those with probable dementia had the highest depressive/anxiety symptoms, with 21.9% exhibiting moderate to severe symptoms, followed by those with possible dementia (14.3% with moderate to severe symptoms) and those with no dementia (6.3% with moderate to severe symptoms). The two dementia groups did not differ in the number of social engagements in the past month; however, both groups had fewer engagements than the no-dementia group. Of the six types of social engagement, the three groups did not significantly differ in religious service attendance, but the lower proportions of the two dementia groups than the no-dementia group visited family/friends, attended clubs or meetings, worked for pay, volunteered, and went out for enjoyment.

**Table 1.** Prevalence of Depressive/Anxiety Symptoms, Social Engagement, and Loneliness, and the Sociodemographic and Other Characteristics by Dementia Status

	All	No dementia (0)	Possible dementia (1)	Probable dementia (2)	*p* among all 3 groups	*p* between (1) & (2)
N (%)	7,547 (100)	6,167 (86.8)	772 (7.7)	608 (5.5)		
Depressive/anxiety symptoms (0–12), M (SE)	1.73 (0.04)	1.59 (0.04)	2.37 (0.14)	3.03 (0.17)	<.001	.002
By symptom severity (%)					<.001	.019
Normal (0–2)	73.0	75.1	62.8	54.2		
Mild (3–5)	19.2	18.6	22.9	23.9		
Moderate/severe (6–12)	7.8	6.3	14.3	21.9		
No. of past-month social engagement (0–6), M (SE)	2.98 (0.03)	3.10 (0.03)	2.25 (0.09)	2.21 (0.09)	<.001	.778
Type of social engagement (%)						
Visits with family/friends	86.1	88.4	70.3	72.0	<.001	.658
Religious service attendance	52.3	53.0	48.8	46.2	.064	.543
Club meeting attendance	38.8	41.4	22.5	21.2	<.001	.668
Work for pay	19.3	20.8	11.1	6.0	<.001	.078
Volunteering	25.0	26.6	14.2	15.1	<.001	.756
Going out for enjoyment	77.0	79.7	57.6	60.9	<.001	.482
Frequency of loneliness (1–5), M (SE)	1.85 (0.01)	1.80 (0.01)	2.09 (0.06)	2.28 (0.05)	<.001	.015
Loneliness frequency in groups (%)					<.001	.061
Never/rarely	76.7	78.5	67.5	60.0		
2–4 days a week	16.2	15.5	19.9	23.1		
5+ days a week	7.1	6.0	12.6	16.9		
Age group (years, %)					<.001	.024
65–74	52.5	54.8	40.6	31.0		
75–84	37.2	36.8	37.4	43.4		
85+	10.3	8.3	22.0	25.6		
Female (%)	55.1	55.7	50.2	51.9	.074	.687
Race/ethnicity (%)					<.001	.270
Non-hispanic white	78.0	80.3	61.8	66.2		
Non-hispanic black	8.6	8.0	12.6	11.5		
Hispanic	8.4	7.2	16.4	16.4		
Other	5.0	4.5	9.2	5.9		
Care facility residence (%)	4.0	3.0	9.2	11.7	<.001	.247
>= Bachelor’s degree (%)	35.7	37.9	22.5	19.4	<.001	.407
Income <$43,000 (%)	37.0	34.7	50.9	53.1	<.001	.610
No. of chronic medical conditions, M (SE)	2.36 (0.02)	2.32 (0.02)	2.54 (0.08)	2.60 (0.10)	.007	.605
Past-month activity-limiting pain (%)	32.1	31.2	38.7	37.4	.007	.749
Social network size (0–5), M (SE)	2.54 (0.02)	2.62 (0.02)	1.99 (0.07)	2.00 (0.07)	<.001	.918

*Note.* Probability values were calculated using Pearson’s χ^2^ tests for categorical variables and ANOVA and t tests for continuous variables.

Those with probable dementia reported the highest frequency of loneliness, followed by those with possible dementia and those with no dementia. In terms of the percentages of those reporting loneliness, 40% of those with probable dementia and 32.5% of those with possible dementia, compared to 23.3% of those with no dementia, reported feeling lonely two or more days a week. Specifically, 16.9%, 12.6%, and 6.0% of individuals with probable dementia, possible dementia, and no dementia, respectively, reported feeling lonely five or more days a week. In these percentages, the two dementia groups were marginally significantly different from each other (*F*[1.92, 107.30] = 2.92, *χ*^2^ [*df* = 2] = 8.77, *p* = .061).

### Sociodemographic and Health Characteristics and Social Network Size by Dementia Status

Table 1 also shows that both dementia groups did not significantly differ from each other in sociodemographic factors (except for older age among those with probable dementia), health status, or social network size. However, both dementia groups significantly differed from the no-dementia group in all other sociodemographic factors (except for gender distribution), health status, and social network size. Compared to the no-dementia group, both dementia groups included higher percentages of racial/ethnic minorities, residents of care facilities, individuals with an income <$43,000, and those experiencing activity-limiting pain, but included a lower percentage of individuals with a college degree. Both dementia groups also had more chronic medical conditions and smaller social networks.

### Mediation Model Results

Table 2 shows the mediation model results: the direct effects of possible and probable dementia, social engagement, and loneliness on depression/anxiety symptoms and the effects of possible and probable dementia on social engagement and loneliness.

**Table 2.** Effects of Social Engagement and Loneliness on the Association Between Dementia Status and Depression/Anxiety Symptoms: Model Parameters From the Mediation Model Using Path Analyses

Model parameter	*B (SE)*	*t*	*p*	95% CI
Depressive/anxiety symptom severity				
Social engagement	−0.17 (0.03)	−5.85	<.001	[−0.22, −0.11]
Loneliness	0.78 (0.05)	16.51	<.001	[0.68, 0.87]
Possible dementia	0.28 (0.14)	2.03	.047	[0.00, 0.55]
Probable dementia	0.79 (0.15)	5.34	<.001	[0.50, 1.09]
Age between 75 and 84	−0.12 (0.07)	−1.57	.121	[−0.27, 0.03]
Age 85 and older	−0.24 (0.10)	−2.44	.018	[−0.43, −0.04]
Female	0.27 (0.06)	4.24	<.001	[0.14, 0.39]
Non-hispanic black	0.06 (0.09)	0.64	.527	[−0.12, 0.24]
Hispanic	0.25 (0.11)	2.32	.024	[0.03, 0.47]
Other race	0.14 (0.15)	0.93	.355	[−0.16, 0.43]
Care facility residence	−0.16 (0.13)	−1.22	.227	[−0.43, 0.10]
≥Bachelor’s degree	−0.16 (0.06)	−2.77	.008	[−0.28, −0.04]
Income <$43,000	0.19 (0.08)	2.49	.016	[0.04, 0.35]
No. of medical conditions	0.16 (0.03)	6.09	<.001	[0.11, 0.22]
Activity-limiting pain	0.96 (0.08)	11.99	<.001	[0.80, 1.12]
Social network size	−0.01 (0.02)	−0.42	.679	[−0.05, 0.04]
Social engagement				
Possible dementia	−0.53 (0.08)	−6.61	<.001	[−0.69, −0.37]
Probable dementia	−0.52 (0.09)	−5.63	<.001	[−0.71, −0.34]
Age between 75 and 84	0.01 (0.04)	0.03	.977	[−0.08, 0.08]
Age 85 and older	−0.31 (0.05)	−5.76	<.001	[−0.42, −0.20]
Female	0.20 (0.04)	5.11	<.001	[0.12, 0.27]
Non-hispanic black	−0.11 (0.04)	−2.74	.008	[−0.20, −0.03]
Hispanic	−0.45 (0.05)	−8.30	<.001	[−0.55, −0.34]
Other race	−0.19 (0.08)	−2.37	.021	[−0.35, −0.03]
Care facility residence	0.02 (0.10)	0.19	.849	[−0.18, 0.21]
≥Bachelor’s degree	0.40 (0.05)	7.48	<.001	[0.29, 0.50]
Income <$43,000	−0.42 (0.05)	−9.00	<.001	[−0.51, −0.33]
No. of medical conditions	−0.08 (0.01)	−5.53	<.001	[−0.11, −0.05]
Activity-limiting pain	−0.22 (0.04)	−5.69	<.001	[−0.30, −0.15]
Social network size	0.13 (0.02)	8.49	<.001	[0.10, 0.16]
Loneliness				
Social engagement	−0.08 (0.01)	−7.41	<.001	[−0.10, −0.06]
Possible dementia	0.17 (0.06)	2.61	.012	[0.04, 0.30]
Probable dementia	0.35 (0.06)	6.32	<.001	[0.24, 0.46]
Age between 75 and 84	−0.08 (0.03)	−2.70	.009	[−0.14, −0.02]
Age 85 and older	−0.01 (0.04)	−0.30	.766	[−0.10, 0.07]
Female	0.21 (0.03)	6.97	<.001	[0.15, 0.27]
Non-hispanic black	−0.11 (0.04)	−2.56	.013	[−0.19, −0.02]
Hispanic	−0.04 (0.05)	−0.96	.342	[−0.14, 0.05]
Other race	−0.02 (0.09)	−0.25	.806	[−0.21, 0.16]
Care facility residence	0.16 (0.08)	1.92	.060	[−0.01, 0.32]
≥Bachelor’s degree	0.01 (0.04)	0.24	.812	[−0.07, 0.09]
Income <$43,000	0.11 (0.04)	3.96	.003	[0.04, 0.19]
No. of medical conditions	0.07 (0.01)	5.49	<.001	[0.04, 0.09]
Activity-limiting pain	0.30 (0.03)	8.65	<.001	[0.23, 0.37]
Social network size	−0.03 (0.01)	−2.25	.029	[-0.06, 0.00]

*Note. N* = 7,537; Population size = 46,376,790; Number of strata = 56; Number of PSUs = 112, Design *df* = 56.

The range of depressive/anxiety symptoms was 0-12; the range for social engagement was 0-6, respectively; and the range for social network size was 0-5. The reference group for Black, Hispanic, and Other was non-Hispanic White.

#### Direct Effects of Dementia, Social Engagement, and Loneliness

The top section of Table 2 shows that depressive/anxiety symptoms were significantly positively associated with possible dementia (*B* = 0.28, *SE* = 0.14, *t* = 2.03, *p* = .047), probable dementia (*B* = 0.79, *SE* = 0.15, *t* = 5.34, *p* < .001), and loneliness (*B* = 0.78, *SE* = 0.05, *t* = 16.51, *p* < .001), but they were significantly negatively associated with social engagement (*B* = −0.17, *SE* = 0.03, *t* = −5.85, *p* < .001). An adjusted Wald test comparing the two path coefficients indicated that the association between depressive/anxiety symptoms and probable dementia was significantly stronger than that for possible dementia (*F*[1,56] = 8.20, *p* = .006). In addition, depressive/anxiety symptoms were negatively associated with ages 85 and older and having a college degree, but they were positively associated with female gender, Hispanic ethnicity, having income <$43,000, having more chronic conditions, and having activity-limiting pain.

The middle and bottom sections in Table 2 show that after adjusting for sociodemographic and health characteristics and social network size, possible and probable dementia were negatively associated with social engagement (*B* = −0.53, *SE* = 0.08, *t* = −6.61, *p* < .001 for possible dementia; *B* = −0.52, *SE* = 0.09, *t* = −5.63, *p* < .001 for probable dementia), and significantly positively associated with loneliness (*B* = 0.17, *SE* = 0.06, *t* = 2.61, *p* = .012 for possible dementia; *B* = 0.35, *SE* = 0.06, *t* = 6.32, *p* < .001 for probable dementia). Adjusted Wald tests indicated no significant difference in the strengths of the associations between social engagement and possible and probable dementia (*F*(1,56) = 0.01, *p* = .921). However, the association between loneliness and probable dementia was significantly stronger than that for possible dementia (*F*(1,56) = 5.16, *p* = .027). Loneliness was also significantly negatively associated with social engagement (*B* = −0.08, *SE* = 0.01, *t* = −7.41, *p* < .001). These results largely support the study’s hypotheses on direct effects.

#### Indirect Effects of Social Engagement and Loneline ss on Depressive/Anxiety Symptoms

The top rows of Table 3 display the bootstrapped indirect effects of possible dementia on depressive/anxiety symptoms through social engagement (0.10, 95% CI [0.07, 0.12], *z* = 7.18, *p* < .001) and loneliness (0.11, 95% CI [0.04, 0.18], *z* = 3.21, *p* = .001). The bottom rows show the corresponding indirect effects for probable dementia on depressive/anxiety symptoms through social engagement (0.10, 95% CI [0.08, 0.13], *z* = 7.13, *p* < .001) and loneliness (0.21, 95% CI [0.13, 0.29], *z* = 5.14, *p* < .001). These results support the study’s hypotheses on mediation effects. Additional bootstrap tests indicated no significant difference in the magnitude of the indirect effects of possible versus probable dementia via social engagement (difference = −0.008, 95% CI [-0.034, 0.016]). However, the indirect effect via loneliness was significantly stronger for probable dementia than for possible dementia (difference = −0.097, 95% CI [-0.199, −0.002]), suggesting that loneliness was a more prominent mediator in the pathway from probable dementia to depressive/anxiety symptoms.

**Table 3.** Indirect Effects of Social Engagement and Loneliness on the Relationship Between Possible and Probable Dementia and Depressive/Anxiety Symptoms

Direct effect^a^ (DE)		Indirect effect^b^ (IE)					Total effect (TE)	Proportion of IE of TE	Ratio of IE to DE
	*B (SE)*		*B (SE)*	*z*	*p*	*95% CI*	DE + IE	IE/TE	IE/DE
Possible dementia	0.28 (0.14)	Social engagement	0.10 (0.01)	7.18	<.001	[0.07, 0.12]	0.38	0.26	0.36
		Loneliness	0.11 (0.04)	3.21	.001	[0.04, 0.18]	0.39	0.28	0.39
Probable dementia	0.79 (0.15)	Social engagement	0.10 (0.01)	7.13	<.001	[0.08, 0.13]	0.89	0.11	0.13
		Loneliness	0.21 (0.04)	5.14	<.001	[0.13, 0.29]	1.00	0.21	0.27

^a^*B(SE)* are from the top section of Table 2.

^b^*B(SE)* and *z* and *p* values are from bootstrapped results.

Table 3 also reports the proportion of each indirect effect relative to the total effect and the ratio of each indirect effect to the corresponding direct effect. For possible dementia, the proportions of the indirect effects via social engagement and loneliness to the total effect on depressive/anxiety symptoms were 0.26 and 0.28, respectively; the corresponding ratios to the direct effect were 0.36 and 0.39. For probable dementia, the proportions of the indirect effects via social engagement and loneliness to the total effect were 0.11 and 0.13, respectively, and the corresponding ratios to the direct effect were 0.13 and 0.27.

## Discussion

In this study, we examined the prevalence of depressive/anxiety symptoms, social engagement, and loneliness among older adults living with dementia, and how social engagement (or the lack of it) and loneliness contribute to their depressive/anxiety symptoms. Our findings show that 13.2% of the Medicare beneficiaries aged 65 and older who were able to self-report their depressive/anxiety symptoms, social engagement, and loneliness had either possible or probable dementia. The prevalence of depressive/anxiety symptoms and loneliness was higher among those with probable than possible dementia, with a little more than one fifth of older adults with probable dementia experiencing moderate to severe depressive/anxiety symptoms and nearly one quarter reporting near-daily loneliness. The two dementia groups had fewer social engagements than the no-dementia group, but the two dementia groups did not significantly differ from each other.

The type of social engagement offers valuable insights. Older adults with and without dementia attended religious services at similar rates. The perceived and actual benefits of religious practice likely motivated both groups to participate. Systematic reviews of religious activities among older adults with dementia show that religion, faith, or spirituality helped them develop coping strategies to accept their condition, sustain relationships, maintain hope, and find meaning in life, which can slow cognitive decline and enhance quality of life (Agli et al., 2015; Daly et al., 2019). However, those with dementia were significantly less likely to engage in other activities, including visiting family and friends. As aforementioned, the lower social engagement in older adults with dementia may result from voluntary withdrawal due to shame, embarrassment, apathy, agitation, and involuntary social exclusion (Birt et al., 2020; Lozupone et al., 2024; Trindade et al., 2023). Reduced social engagement reflects disruptions in broader social and community activities, increasing social isolation. Past studies have also highlighted that a lack of enjoyable activities is a risk factor for declines in both mental and physical health (Bamonti & Fiske, 2021; Ong & Lee, 2023).

The path model confirmed significant direct associations between depressive/anxiety symptoms and both dementia statuses, social engagement, and loneliness, as well as a significant negative association between social engagement and loneliness. While the strength of the association between social engagement and dementia did not differ by dementia status, loneliness was more strongly associated with probable dementia than possible dementia. Bootstrap tests also showed that the indirect pathway through social engagement was comparable across dementia statuses. In contrast, loneliness was a significantly stronger mediator between probable dementia and depressive/anxiety symptoms than for possible dementia, despite lower proportion and ratio metrics for probable dementia. This discrepancy reflects the substantially larger total and direct effects of probable dementia, which reduce the relative contribution of the indirect effect via loneliness. Thus, although its relative share is smaller, loneliness plays a more prominent mediating role in the probable dementia pathway. The relationship between loneliness and depressive/anxiety symptoms may be bidirectional in cross-sectional studies; however, longitudinal research suggests a stronger effect from loneliness to mental health outcomes (Domènech-Abella et al., 2019). Loneliness is also a known risk factor for behavioral and psychological symptoms of dementia (Sun et al., 2021), highlighting the importance of addressing loneliness in dementia care.

Of the covariates, social network size was not significantly associated with depressive/anxiety symptoms but was positively associated with social engagement and negatively associated with loneliness. The directionality of the positive association with social engagement cannot be ascertained in cross-sectional data; however, having more supportive people was likely a motivating factor for engaging in activity, although social engagement may have also facilitated expanding one’s social network. The findings also show that physical and functional health problems played a significant role in depressive/anxiety symptoms, reduced social engagement, and increased loneliness. A previous longitudinal study showed that people with dementia generally had multiple health conditions, and those with more health conditions were lonelier and had poorer quality of life and poorer well-being; however, the number of health conditions had either minimal or no influence on cognition and social isolation over time (Sabatini et al., 2024).

Regarding sociodemographic covariates, our findings indicate that low income (<$43,000) was associated with lower levels of social engagement and higher levels of loneliness and depressive/anxiety symptoms, suggesting that financial insecurity poses a significant barrier to psychosocial well-being in later life. We also found that Hispanic ethnicity was associated with higher depressive/anxiety symptoms, and all minoritized older adults reported significantly lower social engagement; however, this was not associated with greater loneliness. Notably, being Black was linked to a lower likelihood of loneliness, but being Hispanic was no different from being White. Higher levels of depressive symptoms without having a higher level of loneliness among Hispanic older adults may reflect a complex interplay of social support and structural disadvantages. While familism and strong intergenerational ties may protect against loneliness, barriers such as limited access to culturally and linguistically appropriate mental health care, underinsurance, and stigma toward psychological distress may lead to under-recognition or undertreatment of depressive symptoms (Sadule-Rios, 2012). The observed decoupling of lower social engagement from greater loneliness among minoritized older adults might be due to limitations in how social engagement is measured. Conventional indicators, such as participation in clubs, recreational outings, or classes, may not fully capture the culturally salient forms of interaction common in Black and Hispanic older adults. These may include extended family interactions, religious involvement, and multigenerational co-residence. Such informal and family-centered connections may help buffer against loneliness despite limited broader community participation (Taylor & Nguyen, 2020). Further research is needed to explore the complex relationships among social engagement, loneliness, and depressive/anxiety symptoms in minoritized older adults living with dementia.

This study had some limitations related to data constraints. Depressive/anxiety symptoms were assessed using only four items each, loneliness was measured with a single item, and social engagement was not captured in terms of frequency. Since only self-respondents were included, the findings are primarily applicable to individuals with relatively high functioning. Additionally, the cross-sectional nature of the survey data limits the ability to infer causality, and self-reports of depression/anxiety, social engagement, and loneliness may have been affected by recall or social desirability bias. Social networks in NHATS included only those providing emotional support, which may have excluded individuals offering instrumental support to older adults with dementia. As symptoms of dementia can fluctuate over short periods, particularly in terms of mood, behavior, and cognitive clarity, such variability may also influence how older adults experience and report psychosocial constructs such as loneliness and social engagement. Given that the NHATS measures of social engagement and loneliness assess behavior and perception over short reference periods (e.g., “in the past month” and “in the past week,” respectively), these indicators may reflect transient cognitive or emotional states rather than stable patterns. While these short-term measures provide valuable snapshots of lived experiences, they may also introduce measurement variability, particularly among respondents with dementia.

Despite these limitations, this study was the first to examine the interrelationships among dementia, social engagement, loneliness, and depressive/anxiety symptoms in late life. The findings offer valuable insights into the types of potential interventions needed to enhance the emotional well-being and quality of life of older adults living with dementia. Specifically, this study highlights the essential roles of enhancing social engagement and reducing loneliness in improving the mental health of older adults living with dementia. Targeted interventions that promote meaningful engagement, alleviate loneliness, and reduce depressive/anxiety symptoms are needed. Routine clinical assessments should incorporate evaluations of social engagement and loneliness to identify at-risk individuals. Training caregivers to recognize and respond to signs of loneliness and emotional distress is also critical. Although the evidence base remains limited, growing research supports the effectiveness of interventions such as cognitive stimulation, music therapy, and psychological approaches—including cognitive behavioral therapy, pleasant events therapy, and problem-solving therapy—in reducing loneliness and depressive/anxiety symptoms among older adults with dementia (Kishita et al., 2020; Noone et al., 2019; Orrell et al., 2017; Rai et al., 2021). Technology-based interventions, including social robots and multimedia systems, have also shown promise (Budak et al., 2023; Sun et al., 2024). Facilitated small-group activities in settings such as senior centers and other community spaces, and individualized volunteer befriending (Sun et al., 2022) can help foster and preserve social connections among older adults with dementia. Finally, building dementia-friendly communities with accessible public spaces and transportation can further promote social inclusion and community participation (Hung et al., 2021).

## Data Availability

The data used in this study (The National Aging and Health Trends Study) are in the public domain.*

## References

[bibr1-01640275251380531] Agli O. Bailly N. Ferrand C. (2015). Spirituality and religion in older adults with dementia: A systematic review. International Psychogeriatrics, 27(5), 715–725. 10.1017/S1041610214001665 25155440

[bibr2-01640275251380531] Albasheer O. Abdelwahab S. I. Zaino M. R. Altraifi A. A. A. Hakami N. El-Amin E. I. Alshehri M. M. Alghamdi S. M. Alqahtani A. S. Alenazi A. M. Alqahtani B. Alhowimel A. Uddin S. Khalafalla H. E. E. Medani I. E. (2024). The impact of social isolation and loneliness on cardiovascular disease risk factors: A systematic review, meta-analysis, and bibliometric investigation. Scientific Reports*, *14(1), Article 12871. 10.1038/s41598-024-63528-4 PMC11150510 38834606

[bibr3-01640275251380531] Aldridge H. Fisher P. Laidlaw K. (2019). Experiences of shame for people with dementia: An interpretative phenomenological analysis. Dementia, 18(5), 1896–1911. 10.1177/1471301217732430 28958170

[bibr4-01640275251380531] Bamonti P. M. Fiske A. (2021). Engaging in pleasant events explains the relation between physical disability and mental health outcomes in older adults. Aging & Mental Health, 25(2), 225–233. 10.1080/13607863.2019.1683811 31684753

[bibr5-01640275251380531] Bian Z. Xu R. Shang B. Lv F. Sun W. Li Q. Gong Y. Luo C. (2024). Associations between anxiety, depression, and personal mastery in community-dwelling older adults: A network-based analysis. BMC Psychiatry, 24(1), 192. 10.1186/s12888-024-05644-z 38454373 PMC10921593

[bibr6-01640275251380531] Biggs S. Carr A. Haapala I. (2019). Dementia as a source of social disadvantage and exclusion. Australasian Journal on Ageing, 38(Suppl 2), 26–33. 10.1111/ajag.12654 31496064

[bibr7-01640275251380531] Birt L. Griffiths R. Charlesworth G. Higgs P. Orrell M. Leung P. Poland F. (2020). Maintaining social connections in dementia: A qualitative synthesis. Qualitative Health Research, 30(1), 23–42. 10.1177/1049732319874782 31550999

[bibr8-01640275251380531] Botto R. Callai N. Cermelli A. Causarano L. Rainero I. (2022). Anxiety and depression in alzheimer's disease: A systematic review of pathogenetic mechanisms and relation to cognitive decline. Neurological Sciences: Official Journal of the Italian Neurological Society and of the Italian Society of Clinical Neurophysiology, 43(7), 4107–4124. 10.1007/s10072-022-06068-x 35461471 PMC9213384

[bibr9-01640275251380531] Bowirrat A. Elman I. Dennen C. A. Gondré-Lewis M. C. Cadet J. L. Khalsa J. Baron D. Soni D. Gold M. S. McLaughlin T. J. Bagchi D. Braverman E. R. Ceccanti M. Thanos P. K. Modestino E. J. Sunder K. Jafari N. Zeine F. Badgaiyan R. D. Blum K. (2023). Neurogenetics and epigenetics of loneliness. Psychology Research and Behavior Management, 16, 4839–4857. 10.2147/PRBM.S423802 38050640 PMC10693768

[bibr10-01640275251380531] Brzezińska A. Bourke J. Rivera-Hernández R. Tsolaki M. Woźniak J. Kaźmierski J. (2020). Depression in dementia or dementia in depression? Systematic review of studies and hypotheses. Current Alzheimer Research, 17(1), 16–28. 10.2174/1567205017666200217104114 32065103

[bibr11-01640275251380531] Budak K. B. Atefi G. Hoel V. Laporte Uribe F. Meiland F. Teupen S. Felding S. A. Roes M. (2023). Can technology impact loneliness in dementia? A scoping review on the role of assistive technologies in delivering psychosocial interventions in long-term care. Disability and Rehabilitation: Assistive Technology, 18(7), 1107–1119. 10.1080/17483107.2021.1984594 34752177

[bibr12-01640275251380531] Burks H. B. des Bordes J. K. A. Chadha R. Holmes H. M. Rianon N. J. (2021). Quality of life assessment in older adults with dementia: A systematic review. Dementia and Geriatric Cognitive Disorders, 50(2), 103–110. 10.1159/000515317 34167127

[bibr13-01640275251380531] Chen I. W. Sung J. Y. Wang W. H. (2025). The impact of behavioral and psychological symptoms of dementia on mental health, sleep quality, and caregivers’ burden. International Journal of Geriatric Psychiatry*, *40(4), Article e70080. 10.1002/gps.70080 40261108

[bibr14-01640275251380531] Daly L. Fahey-McCarthy E. Timmins F. (2019). The experience of spirituality from the perspective of people living with dementia: A systematic review and meta-synthesis. Dementia, 18(2), 448–470. 10.1177/1471301216680425 27941158

[bibr15-01640275251380531] Defrancesco M. Marksteiner J. Kemmler G. Fleischhacker W. W. Blasko I. Deisenhammer E. A. (2017). Severity of depression impacts imminent conversion from mild cognitive impairment to Alzheimer's Disease. Journal of Alzheimer's Disease: JAD, 59(4), 1439–1448. 10.3233/JAD-161135 28731429

[bibr16-01640275251380531] Domènech-Abella J. Mundó J. Haro J. M. Rubio-Valera M. (2019). Anxiety, depression, loneliness and social network in the elderly: Longitudinal associations from the Irish Longitudinal Study on Ageing (TILDA). Journal of Affective Disorders, 246, 82–88. 10.1016/j.jad.2018.12.043 30578950

[bibr17-01640275251380531] Hackett R. A. Steptoe A. Cadar D. Fancourt D. (2019). Social engagement before and after dementia diagnosis in the English longitudinal study of ageing. PLoS One*, *14(8), Article e0220195. 10.1371/journal.pone.0220195 PMC6675105 31369590

[bibr18-01640275251380531] Holt-Lunstad J. Smith T. B. Baker M. Harris T. Stephenson D. (2015). Loneliness and social isolation as risk factors for mortality: A meta-analytic review. Perspectives on Psychological Science: A Journal of the Association for Psychological Science, 10(2), 227–237. 10.1177/1745691614568352 25910392

[bibr19-01640275251380531] Honda Y. Meguro K. Meguro M. Akanuma K. (2013). Social withdrawal of persons with vascular dementia associated with disturbance of basic daily activities, apathy, and impaired social judgment. Care Management Journals: Journal of case management; The journal of long term home health care, 14(2), 108–113. 10.1891/1521-0987.14.2.108 23930516

[bibr20-01640275251380531] Htun H. L. Teshale A. B. Sun H. Ryan J. Owen A. J. Woods R. L. Shah R. C. Chong T. T. Freak-Poli R. (2025). Changes in loneliness, social isolation, and social support: A gender-disaggregated analysis of their associations with dementia and cognitive decline in older adults. International Journal of Geriatric Psychiatry*, *40(3), Article e70065. 10.1002/gps.70065 PMC11882408 40044457

[bibr21-01640275251380531] Huang W. Zhu W. Chen H. Li F. Huang J. Zhou Y. Sun X. Lan Y. (2022). Longitudinal association between depressive symptoms and cognitive decline among middle-aged and elderly population. Journal of Affective Disorders, 303, 18–23. 10.1016/j.jad.2022.01.107 35108603

[bibr22-01640275251380531] Hung L. Hudson A. Gregorio M. Jackson L. Mann J. Horne N. Berndt A. Wallsworth C. Wong L. Phinney A. (2021). Creating dementia-friendly communities for social inclusion: A scoping review. Gerontology & Geriatric Medicine*, *7, Article 23337214211013596. 10.1177/23337214211013596 PMC8127744 34036118

[bibr23-01640275251380531] Ismail Z. Gatchel J. Bateman D. R. Barcelos-Ferreira R. Cantillon M. Jaeger J. Donovan N. J. Mortby M. E. (2018). Affective and emotional dysregulation as pre-dementia risk markers: Exploring the mild behavioral impairment symptoms of depression, anxiety, irritability, and euphoria. International Psychogeriatrics, 30(2), 185–196. 10.1017/S1041610217001880 28899446

[bibr24-01640275251380531] Jacobson N. C. Newman M. G. (2017). Anxiety and depression as bidirectional risk factors for one another: A meta-analysis of longitudinal studies. Psychological Bulletin, 143(11), 1155–1200. 10.1037/bul0000111 28805400

[bibr25-01640275251380531] Kasper J. D. Freedman V. A. Spillman B. Skehan M. E. Hu M. (2024). Addendum to classification of persons by dementia status in the National Health and Aging Trends Study for follow-up rounds. Johns Hopkins Bloomberg School of Public Health. Available at. https://www.nhats.org/

[bibr26-01640275251380531] King-Kallimanis B. Gum A. M. Kohn R. (2009). Comorbidity of depressive and anxiety disorders for older Americans in the national comorbidity survey-replication. American Journal of Geriatric Psychiatry: Official Journal of the American Association for Geriatric Psychiatry, 17(9), 782–792. 10.1097/JGP.0b013e3181ad4d17 19700950

[bibr27-01640275251380531] Kishita N. Backhouse T. Mioshi E. (2020). Nonpharmacological interventions to improve depression, anxiety, and quality of life (QoL) in people with dementia: An overview of systematic reviews. Journal of Geriatric Psychiatry and Neurology, 33(1), 28–41. 10.1177/0891988719856690 31203712

[bibr28-01640275251380531] Kłosińska U. Leszko M. (2024). Do I have symptoms of dementia: A discursive study of awareness and shame among people with advanced dementia. The Gerontologist, 64(8), gnae067. 10.1093/geront/gnae067 38835189

[bibr29-01640275251380531] Kotwal A. A. Allison T. A. Halim M. Garrett S. B. Perissinotto C. M. Ritchie C. S. Smith A. K. Harrison K. L. (2024). “Relationships, very quickly, turn to nothing”: Loneliness, social isolation, and adaptation to changing social lives among persons living with dementia and care partners. The Gerontologist, 64(4), gnae014. 10.1093/geront/gnae014 38499400 PMC10948338

[bibr30-01640275251380531] Kroenke K. Spitzer R. L. Williams J. B. (2003). The patient health questionnaire-2: Validity of a two-item depression screener. Medical Care, 41(11), 1284–1292. 10.1097/01.MLR.0000093487.78664.3C 14583691

[bibr31-01640275251380531] Kroenke K. Spitzer R. L. Williams J. B. Löwe B. (2009). An ultra-brief screening scale for anxiety and depression: The PHQ-4. Psychosomatics, 50(6), 613–621. 10.1176/appi.psy.50.6.613 19996233

[bibr32-01640275251380531] Lam J. A. Murray E. R. Yu K. E. Ramsey M. Nguyen T. T. Mishra J. Martis B. Thomas M. L. Lee E. E. (2021). Neurobiology of loneliness: A systematic review. Neuropsychopharmacology, 46(11), 1873–1887. 10.1038/s41386-021-01058-7 34230607 PMC8258736

[bibr33-01640275251380531] Lee J. H. Luchetti M. Aschwanden D. Sesker A. A. Strickhouser J. E. Terracciano A. Sutin A. R. (2022). Cognitive impairment and the trajectory of loneliness in older adulthood: Evidence from the Health and Retirement Study. Journal of Aging and Health, 34(1), 3–13. 10.1177/08982643211019500 34027689 PMC8606613

[bibr34-01640275251380531] Lee J. H. Sutin A. R. Hajek A. Karakose S. Aschwanden D. O'Súilleabháin P. S. Stephan Y. Terracciano A. Luchetti M. (2025). Loneliness and cognition in older adults: A meta-analysis of harmonized studies from the United States, England, India, China, South Africa, Mexico, and Chile. Psychological Medicine*, *55, Article e58. 10.1017/S003329172500011X PMC11939032 39973056

[bibr35-01640275251380531] Lee S. L. Pearce E. Ajnakina O. Johnson S. Lewis G. Mann F. Pitman A. Solmi F. Sommerlad A. Steptoe A. Tymoszuk U. Lewis G. (2021). The association between loneliness and depressive symptoms among adults aged 50 years and older: A 12-year population-based cohort study. The Lancet Psychiatry, 8(1), 48–57. 10.1016/S2215-0366(20)30383-7 33181096 PMC8009277

[bibr36-01640275251380531] Lenze E. J. Schulz R. Martire L. M. Zdaniuk B. Glass T. Kop W. J. Jackson S. A. Reynolds C. F. (2005). The course of functional decline in older people with persistently elevated depressive symptoms: Longitudinal findings from the Cardiovascular Health Study. Journal of the American Geriatrics Society, 53(4), 569–575. 10.1111/j.1532-5415.2005.53202.x 15817000

[bibr37-01640275251380531] Leung D. K. Y. Chan W. C. Spector A. Wong G. H. Y. (2021). Prevalence of depression, anxiety, and apathy symptoms across dementia stages: A systematic review and meta-analysis. International Journal of Geriatric Psychiatry, 36(9), 1330–1344. 10.1002/gps.5556 33905138

[bibr38-01640275251380531] Livingston G. Huntley J. Liu K. Y. Costafreda S. G. Selbæk G. Alladi S. Ames D. Banerjee S. Burns A. Brayne C. Fox N. C. Ferri C. P. Gitlin L. N. Howard R. Kales H. C. Kivimäki M. Larson E. B. Nakasujja N. Rockwood K. Mukadam N. (2024). Dementia prevention, intervention, and care: 2024 report of the lancet standing commission. Lancet (London, England), 404(10452), 572–628. 10.1016/S0140-6736(24)01296-0 39096926

[bibr39-01640275251380531] Lozupone M. Dibello V. Sardone R. Altamura M. Bellomo A. Daniele A. Solfrizzi V. Resta E. Panza F. (2024). Social dysfunction and apathy: Transdiagnostic domains in late-life cognitive disorders. Journal of Alzheimer's Disease: JAD, 100(s1), S57–S61. 10.3233/JAD-240556 39031368

[bibr40-01640275251380531] Luchetti M. Aschwanden D. Sesker A. A. Zhu X. O'Súilleabháin P. S. Stephan Y. Terracciano A. Sutin A. R. (2024). A meta-analysis of loneliness and risk of dementia using longitudinal data from >600,000 individuals. Nature. Mental Health, 2(11), 1350–1361. 10.1038/s44220-024-00328-9 39802418 PMC11722644

[bibr41-01640275251380531] MacNeil-Vroomen J. L. Thompson M. Leo-Summers L. Marottoli R. A. Tai-Seale M. Allore H. G. (2020). Health-care use and cost for multimorbid persons with dementia in the national health and aging Trends study. Alzheimer’s and Dementia, 16(9), 1224–1233. 10.1002/alz.12094 PMC9238348 32729984

[bibr42-01640275251380531] Noone D. Stott J. Aguirre E. Llanfear K. Spector A. (2019). Meta-analysis of psychosocial interventions for people with dementia and anxiety or depression. Aging & Mental Health, 23(10), 1282–1291. 10.1080/13607863.2018.1495177 30328711

[bibr43-01640275251380531] Okeke C. C. Ojo A. Ngige O. Amuchie C. C. Obi C. F. Charles O. R. Ekeocha I. R. Konyeme D. C. Idion K. N. Balogun F. O. Onwuchekwa I. Ugo-Ihanetu E. A. Okafor G. S. Ogona D. E. Okoye C. C. Igboanugo S. A. (2025). A systematic review of the impact and outcome of loneliness and self-isolation on individuals living with cardiovascular disease. Cureus*, *17(3), Article e81277. 10.7759/cureus.81277 PMC12032856 40291206

[bibr44-01640275251380531] Oken B. S. Kaplan J. Klee D. Gallegos A. M. (2024). Contributions of loneliness to cognitive impairment and dementia in older adults are independent of other risk factors and alzheimer's pathology: A narrative review. Frontiers in Human Neuroscience*, *18, Article 1380002. 10.3389/fnhum.2024.1380002 PMC11169707 38873650

[bibr45-01640275251380531] Ong A. D. Lee S. (2023). Variety in pleasant activities is associated with improved mental health: Evidence from two national samples of U.S. Adults. Affective Science, 5(2), 90–98. 10.1007/s42761-023-00225-x 39050039 PMC11264508

[bibr46-01640275251380531] Orrell M. Yates L. Leung P. Kang S. Hoare Z. Whitaker C. Burns A. Knapp M. Leroi I. Moniz-Cook E. Pearson S. Simpson S. Spector A. Roberts S. Russell I. de Waal H. Woods R. T. Orgeta V. (2017). The impact of individual cognitive stimulation therapy (iCST) on cognition, quality of life, caregiver health, and family relationships in dementia: A randomised controlled trial. PLoS Medicine*, *14(3), Article e1002269. 10.1371/journal.pmed.1002269 PMC5369684 28350796

[bibr47-01640275251380531] Penninkilampi R. Casey A. N. Singh M. F. Brodaty H. (2018). The association between social engagement, loneliness, and risk of dementia: A systematic review and meta-analysis. Journal of Alzheimer's Disease: JAD, 66(4), 1619–1633. 10.3233/JAD-180439 30452410

[bibr48-01640275251380531] Perlman D. Peplau L. A. (1984). Loneliness research: A survey of empirical findings. In Peplau L. A. Goldstein S. (Eds.), Preventing the harmful consequences of severe and loneliness (pp. 13–45). National Institute of Mental Health. U.S. Government Printing Office. Chapter 2. https://peplau.psych.ucla.edu/wp-content/uploads/sites/141/2017/07/Perlman-Peplau-84.pdf

[bibr49-01640275251380531] Rafnsson S. B. Orrell M. d’Orsi E. Hogervorst E. Steptoe A. (2020). Loneliness, social integration, and incident dementia over 6 Years: Prospective findings from the English longitudinal study of ageing. Journals of Gerontology Series B: Psychological Sciences and Social Sciences, 75(1), 114–124. 10.1093/geronb/gbx087 28658937 PMC6909434

[bibr50-01640275251380531] Rai H. K. Schneider J. Orrell M. (2021). An individual cognitive stimulation therapy app for people with dementia and carers: Results from a feasibility randomized controlled trial (RCT). Clinical Interventions in Aging, 16, 2079–2094. 10.2147/CIA.S323994 35221680 PMC8866989

[bibr51-01640275251380531] Sabatini S. Martyr A. Hunt A. Gamble L. D. Matthews F. E. Thom J. M. Jones R. W. Allan L. Knapp M. Victor C. Pentecost C. Rusted J. M. Morris R. G. Clare L. (2024). Comorbid health conditions and their impact on social isolation, loneliness, quality of life, and well-being in people with dementia: Longitudinal findings from the IDEAL programme. BMC Geriatrics, 24(1), 23. 10.1186/s12877-023-04601-x 38182985 PMC10768096

[bibr52-01640275251380531] Sadule-Rios N. (2012). A review of the literature about depression in late life among Hispanics in the United States. Issues in Mental Health Nursing, 33(7), 458–468. 10.3109/01612840.2012.675415 22757598

[bibr53-01640275251380531] Shen C. Rolls E. T. Cheng W. Kang J. Dong G. Xie C. Zhao X. M. Sahakian B. J. Feng J. (2022). Associations of social isolation and loneliness with later dementia. Neurology, 99(2), e164–e175. 10.1212/WNL.0000000000200583 35676089

[bibr54-01640275251380531] Singleton D. Mukadam N. Livingston G. Sommerlad A. (2017). How people with dementia and carers understand and react to social functioning changes in mild dementia: A UK-based qualitative study. BMJ Open*, *7(7), Article e016740. 10.1136/bmjopen-2017-016740 PMC5541577 28706105

[bibr55-01640275251380531] Spitzer R. L. Kroenke K. Williams J. B. Löwe B. (2006). A brief measure for assessing generalized anxiety disorder: The GAD-7. Archives of Internal Medicine, 166(10), 1092–1097. 10.1001/archinte.166.10.1092 16717171

[bibr56-01640275251380531] Sun W. Bartfay E. Smye V. Biswas S. Newton D. Pepin M. Ashtarieh B. (2022). Living well with dementia: The role volunteer-based social recreational programs in promoting social connectedness of people with dementia and their caregivers. Aging & Mental Health, 26(10), 1949–1962. 10.1080/13607863.2021.1950614 34353187

[bibr57-01640275251380531] Sun W. Gabel G. Akhter R. Lawson L. Plishewsky J. (2024). Feasibility and acceptability of virtual programs for people with dementia and their caregivers. BMC Geriatrics, 24(1), 783. 10.1186/s12877-024-05375-6 39322955 PMC11423503

[bibr58-01640275251380531] Sun W. Matsuoka T. Oba H. Narumoto J. (2021). Importance of loneliness in behavioral and psychological symptoms of dementia. International Journal of Geriatric Psychiatry, 36(4), 540–546. 10.1002/gps.5450 33091165

[bibr59-01640275251380531] Sundström A. Adolfsson A. N. Nordin M. Adolfsson R. (2020). Loneliness increases the risk of all-cause dementia and alzheimer's disease. Journals of Gerontology Series B: Psychological Sciences and Social Sciences, 75(5), 919–926. 10.1093/geronb/gbz139 31676909 PMC7161366

[bibr60-01640275251380531] Taylor H. O. Nguyen A. W. (2020). Depressive symptoms and loneliness among Black and White older adults: The moderating effects of race. Innovation in Aging, 4(5), igaa048. 10.1093/geroni/igaa048 33354628 PMC7739884

[bibr61-01640275251380531] Trindade P. G. E. Johannessen A. Baptista M. A. T. Maffiolletti V. Dourado M. C. N. (2023). *'*I do not enjoy too much being with people, it takes me a long time to interact*'*: A qualitative analysis of awareness of relationships in people with dementia. Aging & Mental Health, 27(6), 1120–1126. 10.1080/13607863.2022.2084503 35848168

[bibr62-01640275251380531] Vernooij-Dassen M. Verspoor E. Samtani S. Sachdev P. S. Ikram M. A. Vernooij M. W. Hubers C. Chattat R. Lenart-Bugla M. Rymaszewska J. Szczesniak D. Brodaty H. Welmer A. K. Maddock J. van der Velpen I. F. Wiegelmann H. Marseglia A. Richards M. Melis R. Wolf-Ostermann K. (2022). Recognition of social health: A conceptual framework in the context of dementia research. Frontiers in Psychiatry*, *13, Article 1052009. 10.3389/fpsyt.2022.1052009 PMC9798783 36590639

[bibr63-01640275251380531] Victor C. R. Rippon I. Nelis S. M. Martyr A. Litherland R. Pickett J. Hart N. Henley J. Matthews F. Clare L. IDEAL Programme Team . (2020). Prevalence and determinants of loneliness in people living with dementia: Findings from the IDEAL programme. International Journal of Geriatric Psychiatry, 35(8), 851–858. 10.1002/gps.5305 32281151

[bibr64-01640275251380531] Wang S. Molassiotis A. Guo C. Leung I. S. H. Leung A. Y. M. (2023). Association between social integration and risk of dementia: A systematic review and meta-analysis of longitudinal studies. Journal of the American Geriatrics Society, 71(2), 632–645. 10.1111/jgs.18094 36307921

[bibr65-01640275251380531] Wilson S. J. Woody A. Padin A. C. Lin J. Malarkey W. B. Kiecolt-Glaser J. K. (2019). Loneliness and telomere length: Immune and parasympathetic function in associations with accelerated aging. Annals of Behavioral Medicine: A Publication of the Society of Behavioral Medicine, 53(6), 541–550. 10.1093/abm/kay064 30107521 PMC6499407

[bibr66-01640275251380531] Xiang X. An R. (2015). The impact of cognitive impairment and comorbid depression on disability, health care utilization, and costs. Psychiatric Services, 66(11), 1245–1248. 10.1176/appi.ps.201400511 26073413

[bibr68-01640275251380531] Yin J. John A. Cadar D. (2024). Bidirectional associations of depressive symptoms and cognitive function over time. JAMA Network Open*, *7(6), Article e2416305. 10.1001/jamanetworkopen.2024.16305 PMC11167501 38861255

[bibr69-01640275251380531] Zhu Y. Li C. Wu T. Wang Y. Hua R. Ma Y. Xie W. (2023). Associations of cumulative depressive symptoms with subsequent cognitive decline and adverse health events: Two prospective cohort studies. Journal of Affective Disorders, 320, 91–97. 10.1016/j.jad.2022.09.128 36183825

